# Scientific production and technological production: transforming a scientific paper into patent applications

**DOI:** 10.1590/S1679-45082013000100003

**Published:** 2013

**Authors:** Cleber Gustavo Dias, Roberto Barbosa de Almeida

**Affiliations:** 1 Hospital Israelita Albert Einstein, São Paulo, SP, Brazil; 2 Empresa Brasileira de Pesquisa Agropecuária, Brasilia, DF, Brazil

**Keywords:** Organizational innovation, Technological development, Scientific and technical publications, Patents

## Abstract

Brazil has been presenting in the last years a scientific production well-recognized in the international scenario, in several areas of knowledge, according to the impact of their publications in important events and especially in indexed journals of wide circulation. On the other hand, the country does not seem to be in the same direction regarding to the technological production and wealth creation from the established scientific development, and particularly from the applied research. The present paper covers such issue and discloses the main similarities and differences between a scientific paper and a patent application, in order to contribute to a better understanding of both types of documents and help the researchers to chose and select the results with technological potential, decide what is appropriated for industrial protection, as well as foster new business opportunities for each technology which has been created.

## RESUMO

O Brasil vem apresentando, nos últimos anos, uma produção científica bastante reconhecida no cenário internacional, nas mais diversas áreas do conhecimento, tomando por base o impacto de suas publicações em eventos de grande monta e, especialmente, em revistas indexadas de grande circulação. De outra forma, o país parece não caminhar na mesma direção quando se trata da produção tecnológica e da geração de riqueza a partir do desenvolvimento científico estabelecido e, particularmente, a partir da pesquisa científica aplicada. O presente trabalho abordou tal questão e discorreu acerca das principais semelhanças e diferenças entre um artigo científico e o texto de um pedido de patente, a fim de contribuir para um melhor entendimento dos dois tipos de documento, auxiliar os pesquisadores na escolha e seleção dos resultados com potencial tecnológico, decidir o que é adequado para fins de proteção industrial, bem como alavancar novas oportunidades de negócio para cada tecnologia criada.

## INTRODUCTION

Over the last few years, Brazil has played a role as one of the primary players on the stage of international scientific production.

The influential presence of Brazilian researchers at congresses and in indexed journals shows that the country has advanced exponentially on the world scene regarding so-called impact scientific research.

In an interview published by the Regional Council of Medicine of the State of São Paulo (CREMESP)^(1)^, the Research Vice-Principal for Research of the Universidade de São Paulo (USP), Dr. Marco Antônio Zago revealed that qualified scientific production in Brazil, which is published in journals of international research, represented, during the year 2008, about 1.9% of the world total.

A few recent numbers^(2)^ have further highlighted the condition of a nation in full development, as to the quantity of articles currently published by our researchers throughout the world. Additionally, Dr. Zago pointed out during the same interview that the number of scientists was increasing during the year 2008, besides the fact that the investment in Brazil in terms of science and technology also increased significantly, representing, during that year, a value of about 1% of the national Gross Domestic Product (GDP).

Material recently published by Sennes and Britto Filho^(2)^ corroborate the fact that the scientific production of Brazil has been experiencing quantitative progress over the last few years, even though its qualitative advancement has been less expressive.

The same material points out^(2)^ that between the years 1996 and 2005, only 26 articles, in the most diverse areas of knowledge, achieved more than 200 citations. On the other hand, still concerning the year 2008, such as is presented in the abovementioned interview published by CREMESP^(1)^, once again the need for Brazilian researchers to be involved in research and development in companies as a form of transforming scientific development into economic growth, resulting from the transfer of knowledge and technology to the productive sector was highlighted.

In this sense, and as an example, it is worth mentioning data collected and presented in the publication put out by Amadei and Torkomian^(3)^, which analyzed the patent applications of public universities of the State of São Paulo at the National Institute of Industrial Property (INPI).

Additional data, presented by Amadei and Torkomian^(3)^ and reproduced on table 1, revealed the relation between the number of patent applications and the quantity of existing graduate level programs at each university during the period of 2000 to 2006.

**Table 1:** Patent application / graduate program ratio

Institution	Mean number of graduate programs (2000-2006)	Accumulated patents placement (2000-2006)	Placement by program
USP	217.86	128	0.59
UNESP	97.14	45	0.46
UNICAMP	63.43	327	5.16
UFSCAR	18.57	27	1.45
UNIFESP	40.86	21	0.51

Source: modified from Amadei JR, Torkomian AL. As patentes nas universidades: análise dos depósitos das universidades públicas paulistas. Ci Inf. 2009; 38(2):9-18^(3)^.

USP: *Universidade de São Paulo*; UNESP: *Universidade Estadual Paulista* “*Júlio de Mesquita Filho*;” UNICAMP: *Universidade Estadual de Campinas;* UFSCAR: *Universidade Federal de São Carlos*; UNIFESP: *Universidade Federal de São Paulo*.

The results presented in the mentioned publication^(3)^ also showed a relation between the number of patent applications and the number of publications promoted by the institutions of USP, Universidade Estadual de Campinas (UNICAMP), Universidade Estadual Paulista “Júlio de Mesquita Filho” (UNESP), Universidade Federal de São Paulo (UNIFESP) and Universidade Federal de São Carlos (UFSCAR), during the period of 1998 to 2002. It can be clearly noted that even at that time, there was an accentuated distance in the relation between scientific production and technological production, at least when using as reference the number of patent applications. As an example, USP presented, during the cited period, a ratio near 324, that is, for a patent application made by USP, there were more than 300 publications linked to their graduate programs.

A current reflection on the theme^(4)^ addresses the primary factors that lead to a low rate of patents granted to technology developed at teaching institutions, and particularly, those developed at the Graduate Program in Electrical Engineering and Industrial Informatics (CPGEI) at the Universidade Tecnológica Federal do Paraná (UTFPR).

Among the results presented^(4)^, it is important to highlight that only a small percentage of the researchers reported good or sufficient knowledge about the patent systems, besides the fact that most were not aware of the Innovation Law No. 10,973^(5)^.

Nevertheless, the same article^(4)^ showed that a portion of researchers reported the recently developed research projects would have the possibility of intellectual protection, according to their understanding, even though they had asserted that they still did not know, in detail, the process for patent application at the National Institute of Industrial Property (INPI), or due to the fact of not clearly understanding the details for writing and preparing the text for a patent.

Based on prior results, it is possible to affirm, at least as a starting point, that lack of knowledge about the entire process of industrial production on the part of researchers, contributes significantly not only to the low volume of patent applications, but also to the reduced generation of foreign currency as to the technology created and produced in Brazil.

It is evident that scientific publication acts as a reflection of the work developed in national laboratories and may be considered today as one of the drivers for production of patents in the country. Academically acclaimed institutions are currently considerable candidates for applications not only at INPI, as the data already presented here shows, but also abroad, thus contributing towards fostering the establishment of a technological culture in the country, in addition to increasing the added-value of products made in the Brazilian industrial complex.

It is a fact that there is a series of discussions and beliefs that comprehension and use are conflicting goals in the area of research, in other words, basic and applied research are totally separate categories^(6)^. Many times, such a difference in opinion can lead to distortions, even when there is clearly, in a given project, a correlation between science and technology.

The data and results presented by Moura^(7)^ highlight that the area of biotechnology in Brazil has been offering an important interaction between science and technology, in such a way that researchers in this field of knowledge act also as inventors, thus producing both scientific and technological publications.

On the other hand, the same study^(7)^ ratifies that the greatest collaboration in scientific and technological development still occurs, at least during the period evaluated by the article referred to in the area of biotechnology, among federal and state public universities, such as USP, UNICAMP, UNESP and the Universidade Federal do Rio de Janeiro (UFRJ), and research institutions, such as the Oswaldo Cruz Foundation (FIOCRUZ), Butantan Institute, and the Brazilian Agricultural Research Corporation (EMBRAPA). Within this context, it is clear that there is no effective collaboration among such universities and research organizations and the productive sector, reinforcing, once again, the fact that there is yet another factor contributing to the low number of applications, namely, the distance between the teaching and research institutions and the companies in the country, even though this phenomenon has been much discussed, over the last few years, by several players / authors in Brazil.

In this way, regardless of the methods used for evaluation of productivity of Brazilian researchers carried out by development agencies, such as the Coordination for the Improvement of Higher Education Personnel (CAPES), the National Council for Scientific and Technological Development (CNPq), and the Research Support Foundation of the State of São Paulo (FAPESP), currently there is a confirmed fact: Brazilian scientific production grows at an accelerated rate. It is interesting to note that, although it represents an important part of patent applications made at the INPI and Brazilian patent applications performed in the USA (United States Patent and Trademark Office – USPTO) as well as at the World Organization of Intellectual Property (WIPOI), the patent applications resulting from research institutions do not grow in the same proportion.

Considering that, different from what was true in the past, debates as to the distance between the industry and the university have increased significantly, which contributes to more new partnerships, the lack of patent-related culture of our researchers may, in fact, be one of the possible explanations for the small number of patent applications in Brazil^(4)^.

Within this context, the present article presented some similarities and differences between the production of a scientific publication and the text of a patent application, which stand out the main aspects that permeate each category of the documents and contribute to a better understanding, not only on the part of the researchers that transit in both areas, as well as of those who act as intermediaries within this process. Among these intermediaries are professionals and specialists in the area of patents, usually working at innovation centers or hubs, as well as in law firms specialized in industrial property protection.

Regarding this latter topic, it is believed that greater knowledge of researchers and other professionals about the patent system, and in particular, about preparing appropriate documents, may provide a better evaluation of the potential of each new technology created, thus enhancing the possibilities of developing products in partnership, besides new opportunities for its future licensing and/or marketing.

### General structure of a scientific document and a patent document

**Figure 1:**
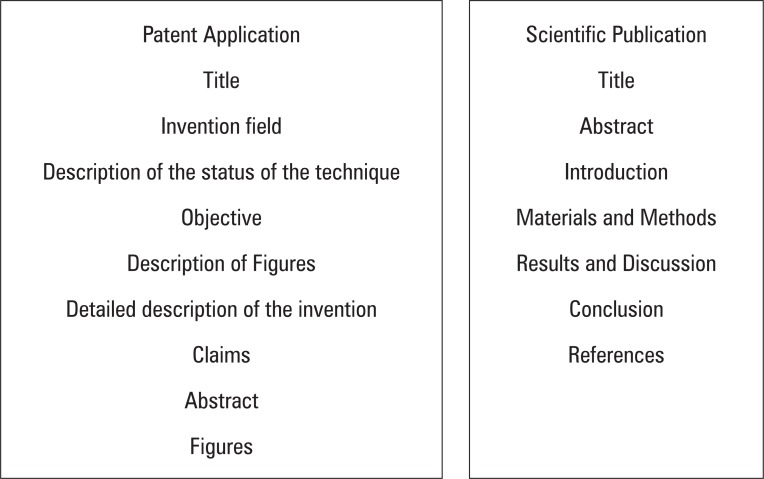
General structure of a patent document and of a scientific publication

With the purpose of providing a general view of the primary differences between a patent application and a scientific publication, initially we present a structural comparison between the two types of document (Figure 1). The structure of the documents described herein, despite not corresponding to a fixed rule, may be used as a general guideline to prepare the respective texts.

Starting with the publication and, in particular, by its title, for the presentation of a scientific article, generally resulting from a Master's degree dissertation or of a Doctorate thesis, the title should name the theme of the research, per se; in other words, indicate by its name, the subject of the proposed paper^(8)^. The abstract, on the other hand, consists of a concise presentation of the content of the scientific article, and aims to offer the reader a complete idea of the content of the document to be analyzed.

The “Introduction” of a scientific publication is commonly designed to announce how the theme will be explored throughout the document text.

Additionally, when this is the case, the introduction raises the state of the issue, revealing what has already been written about the theme and signaling the importance and the interest of the article^(8)^. At this point, it is essential to indicate, as explained by Severino^(8)^, that “in reading the introduction, the reader of the document should feel enlightened about the content of the problem of the article topic, as well as the nature of the type of rationale to be developed.”

The “Materials and Methods” field describes the primary development stages or phases of the article, as well as a list of materials, inputs, equipment, and information systems, among others, used throughout the scientific investigation process.

The “Conclusion” of a scientific article explores the principal results obtained during the research, and outlines the most relevant reflections regarding the data in comparison to the expected and proposed objectives.

Lastly, the “References” place the reader of the article as to the state-of-the-art of the theme investigated. In other words, they support the problem investigated and offer the reader an appreciation of the scientific scenario for the topic under study.

Observing figure 1, a few similarities can be seen between the abovementioned article and a text for patent request.

In synthesis, it is easy to visualize that the text of a patent document has a title, as does an article, besides the objectives, description of the status of the invention technique, and abstract, similar to the objectives and status of the technique contained in the “Introduction” item of a scientific publication and its abstract.

On the other hand, despite the similarities, both documents show some basic differences in the way in which the project or invention are technically addressed. Understanding these differences may help the researcher who is trained to think scientifically and dominates the structure of a scientific publication and desires to transform, when this is the case, his/her work into a patent request. Below, with the help of structures mentioned in figure 1, the primary divergent and convergent points are identified, between a patent application and a scientific article.

### The logical sequence – the problem to be solved

The sequence of thoughts that dominates the text of a patent request and a scientific publication basically diverge as per the highest degree of autonomy presented by the publication, in comparison with the greatest objectivity of a patent application.

According to the Normative Act 127^(9)^, item 15.1.2, which regulates the Law of Industrial Property (LPI) 9,279/96^(10)^, the descriptive report of a patent should “describe, in a clear, concise and precise manner, the solution proposed for an existing problem, as well as the advantages of the invention to be protected relative to the status of the technique.”

Within this logical sequence, the problem to be solved and the solution found for it should be clear. This is the pathway taken by a patent application. To maintain the clarity of the document in the description of the application utilizing this sequence, the problem to be solved must be, initially, well delimited and for this, indicative categories are used, such as, for example, equipment, process, product, or use.

Scientific production does not always originate from the assumption that there is a problem to be solved, but rather that there is something to be revealed which has not been apparent up until then. The revelation of something not yet described generates new information, which is the basis for new interpretation, closing a virtual circle which, to a large extent, contributes to generation of new knowledge. However, the simple fact of revealing something new is not sufficient for a publication to become a patent application.

It is important to point out that, discoveries, mathematical methods, scientific theories, as well as data resulting from experiments, as per article 10 of LPI 9,279/96^(10)^, are not patentable, and thus, despite representing a considerable percentage of scientific publications, cannot be directly converted into patent applications.

Along the line of thinking of a scientific article, the solution of a problem within the specific categories of the equipment, process, product, or use, many times does not necessarily appear as a consequence of the main project. The fact is that, in many cases, a scientific article has a goal that is not the objective resolution of an existing problem, especially when it is a case of pure basic research and not applied research^(6)^.

This is due, in part, to the distance between the university and the productive media, i.e., the Brazilian universities are still not sought out very often when there is a need for proposals of solutions for technological problems. The increased presence of the university by means of proximity with the industry will result, over time, in a greater integration between the two sectors and consequently, in the appearance of research focused on the resolution of real existing problems.

### Identification of similar and/or prior technologies

As to the items “Description of the status of the technique” of a patent request, and the “Introduction” of a scientific publication, the great similarity lies in the fact that both documents report the current stage of development of the subject which is the object of the publication or of protection by patent request^(8,9)^. A scientific publication, when it reports productions prior to the theme under study, identifies, in the so-called “state-of-the-art” or “state-of-knowledge”, mapping of the academic production as to the topic of interest^(11)^ and, as has already been mentioned, the introduction of the scientific article raises such a state of development, revealing what has already been written and discussed about the theme^(8)^.

The patent application, on the other hand, should, as established in the Normative Act 127^(9)^, item 15.1.2, paragraph d, describe the status of the technique that can be considered useful to understanding, searching and examining the invention, reporting, whenever possible, the documents that reflect it and pointing out the existing technical problems.

This difference of needs between a publication and a patent interferes directly in search for documents performed for the characterization of the current status of development of the object of publication/ invention.

Searches for prior documents performed with the intention of confirming the originality of a patent application should be performed after clear identification of the involved inventive concept; whereas, in the case of scientific articles, the search is made, necessarily, for the innovation to be revealed, which in some cases, may be characterized as an invention. At this point, it is important to remember that an invention, in order to be considered as such, should fulfill the requirements of novelty, innovative activity, and industrial application^(12,13)^.

As is established in the current law of industrial property - LPI 9,279/96^(10)^,more specifically in its article 11, an invention is considered new when it is not seen in the status of the technique.

Additionally, it is worth mentioning that article 13 of the same LPI^(10)^ defines that an invention includes inventive activity whenever, for a technician in the subject, who has at least a median level of experience and knowledge, it does not result in an evident or obvious way from the status of the technique.

In this last case, the patent examiner commonly evaluates if a given invention, object of an application, shows technical benefits and advantages sufficient to characterize its inventive activity in light of the documents found in the search of prior documents.

The current LPI^(10)^ also establishes, in article 15, that invention is considered susceptible to industrial application when it may be utilized or produced in any kind of industry, in other words, on a large scale in the productive environment.

Additionally, it is worth mentioning that the invention can, in many cases, be a small part of a scientific production which, if not followed in detail or if underestimated, may be lost within the publication. The false notion that searches for prior examples for patent applications should be directed towards commercially available products comes from the fact that, many times, the holder of a request understands that only technology available on the market may be considered impeditive for granting a new patent.

On the other hand, it is important to point out that article 11 of the LPI^(10)^, first paragraph, defines “The status of the technique is constituted by all that has been made accessible to the public before the date of the patent application, by written or oral description, by use or any other means, in Brazil or overseas, exempting what is set forth in articles 12, 16, and17”.

### Justification of the invention

The concept of justification of the invention is many times used when the structure of academic thinking is transferred to a patent request. The logical structure of a researcher, i.e., that his/her work adds something to the status of the technique and that this addition should be shown within on-going rationale, based on the tendency followed by the last publications, is often followed when a patent request is written with the same line of reasoning as that of a scientific publication.

Although the invention may follow certain master guidelines determined by prior publications, the concepts of invention and patentability pass through the principle of non-obviousness of the material to be protected, also known as the inventive activity already previously described.

The so-called “non-obviousness” is not the concern that exists in preparing a scientific publication, since, as per academic thinking, the next step, despite perhaps being obvious, has not yet been given, and is, therefore, worthy of publication.

For a better understanding, examples may be drawn, such as the identification and characterization of lipid oxidation enzymes present in some plants. These enzymes, called lipoxygenases, are found in several vegetable sources, and they were isolated in soybean and barley and characterized by means of methods that use equipment commonly employed in biotechnology laboratories, such as high-performance liquid chromatography (HPLC) and electrophoresis.

Nevertheless, due to marketing reasons or simply because of the lack of interest of researchers, these enzymes have not yet been characterized in plants that are usually not explored commercially, generating a possibility of research to be developed, which would enable the appearance of new scientific publications. Many times, articles considered repetitive and preliminarily judged as having obvious results, reserve some surprises, which if duly perceived and well interpreted, may open the way for an invention.

Along this line of reasoning, i.e., of the concept of invention, it is worth highlighting that many times, even today, there is confusion about the potential inventive activity of a technological development, due to the technical effect it affords, such as described by the Instituto Dannemann Siemsen de Estudos de Propriedade Intelectual (IDS)^(12)^and reproduced here: “…in the presence of the 1971 Code it was common to misunderstand “inventive activity” with “new or different technical effect”, due to article 9, paragraph e^68^, which defined as non-patentable, as inventions, juxtapositions of known processes, means, or agencies, or a simple change in shapes, proportions, dimensions, or materials, except if this results, as a whole, in a new or different effect.”

Considering what was exposed, it is highly recommended to analyze carefully if a given technological development does not represent a mere juxtaposition of parts, since in this case, there will be a non-negligible chance of INPI questioning its patentability.

### Experimental results

Generally, as a consequence of experimental results, scientific articles have, in these results, the principal justification for existing. Figure 1 shows that the publication contains a field specifically designated for reporting the results obtained during the process of scientific investigation.

Based on these results, theories are often confirmed or contested, new concepts appear and science advances. However, the transfer of this scale of importance of the experimental result to technological production is an error frequently made, in the transformation of a scientific publication to a patent request.

The presentation in a patent application, of experimental results, has the purpose of increasing credibility of the invention described, however, and this has to be well understood. The demonstration or not of these experimental results is not a determining factor for obtaining the patent document. The official agencies of industrial property responsible for granting or not the patent document do not have the task of attesting if the experimental results are suitable, but rather, if the invention treated by the patent fulfills the necessary conditions of patentability, i.e., novelty, inventive activity, and industrial application. The experimental results, when available, are welcome and contribute to confirm the utility of the invention; however, they do not necessarily need to be a part of the patent application.

The advantages proclaimed by the invention in a patent text will, in practice, pass through a much more severe filter than the simple confirmation of good repute of the experimental results presented. This screening happens at the moment of use and/or marketing, by third parties, of the inventive concept described in the patent application or in the patent granted. At this moment, non-confirmation of the advantages described by the invention may result in the inutility of the document, and therefore, in the conclusion that the investment made was in vain.

### Details and scope of protection of a patent request

Scientific publications, in reference to the definition of experimental parameters (usually demonstrated in materials and methods), are generally very timely. Additionally, more comprehensive comments and conclusions regarding the experimental results are frequently made with caution. Nevertheless, when changing a scientific publication into a patent application, these parameters should be interpreted as protection definers of the future letter patent.

Within this context, the more comprehensive the parameters to be protected for a given invention, the greater the scope of protection of the future patents, and consequently, the greater the possibility of avoiding that third parties copy or reproduce the invention. Along this line of thinking, one limiting factor cannot be forgotten, which is the increased probability of the invention already having been anticipated or expected. The greater likelihood of anticipation is proportional to the amplitude of the parameters defined by the invention. Such parameters should be chosen very carefully by the future applicant, in order to provide a careful preparation of the text as to the set of claims and to define what the product object of protection is, in fact.

In this way, it is important to stress the scope of protection of a patent application, defined based on its set of claims, is exactly the portion orientated towards protecting a given technology of interest for the market, and especially for its respective applicant. Figure 1 shows that the patent application should include the “Claims” field, for the very purpose of defining the object to be protected.

It can also clearly be seen, by figure 1, that such a field is not a part of the coverage of a scientific publication, based on its nature, as previously described.

**Figure 2:**
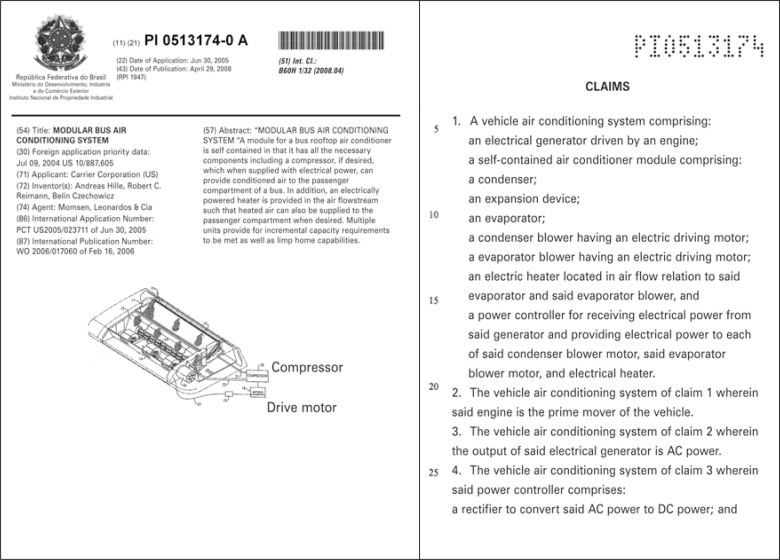
Cover and claims page of a patent application in the electrical area

**Figure 3:**
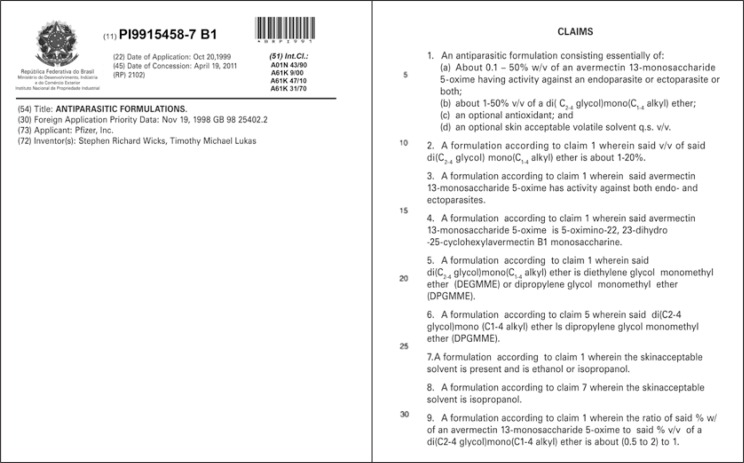
Cover and claims page of a patent application in the chemical/pharmaceutical area

As examples, figures 2 and 3, below, illustrate the cover of two patent applications, in the electrical and chemical areas, respectively, as well as the material to be presented in the part of its claims.

As provided in the article 41 of Law 9,279/96^(10)^, the extension of protection conferred by a patent is determined by the content of its claims, as has already been mentioned, not forgetting, however, that the material defined in the claims will be interpreted based on the descriptive report, especially based on the objectives and detailed description of the invention, as well as with the help of the figures, as is identified in the structure of the document in figure 1.

In this way, it is also important to point out that the article of the cited Law is based on a worldwide practice regarding the fact that the scope of protection sought by means of a patent application is defined by its claims, i.e., it is the text of claims that determines the limits of the rights assured by the patent, and at this point, the parameters chosen for an invention are essentially for the definition of the object to be protected.

### Examples of preferred implementations

Along with the presentation of experimental results, the scientific publication, when it has the objective of obtaining an optimum point of the result of the article presented, describes in details the parameters for such a point. These parameters, despite being timely, should be utilized in the patent application, in item “Detailed description of the invention”, as examples of the preferred implementation of the technology that is the object of protection.

Article 24 of LPI^(10)^ corroborates the declaration, above, in that it defines: “The report should describe the object in a clear and sufficient manner, in order to enable it to be implemented by a technician in the subject and to indicate, when this is the case, the best form of execution.”

Therefore, the implementations of an invention, described and suggested in the patent application, should offer alternatives for the implementation of the technology to be protected, from its primary characterization until new possibilities of construction. As an illustration, the example can be given that a certain patent application makes reference to an invention configured as an actuator, when it is primarily of the electrical type for moving a given piece in a machine, but the same document may also cite, as alternative implementations, a configuration of an electropneumatic or electrohydraulic actuator capable of performing the same function. Another example, while yet within this context, in the case of protection of a pharmaceutical composition containing specific preferential excipients and an active compound (active principle). The same document further mentions as alternative implementations similar excipients that can successfully substitute those cited and preferential, thus increasing the scope of protection of the invention.

### Final considerations

The present paper dealt with the current scenario of national scientific production, in face of the low number of patent applications recorded over the last few years in the country and especially, of the main aspects related to the text of a patent application when compared to a scientific publication. To a certain extent, the structures of both categories of documents were compared, standing out the similarities and the differences observed between the two, as well as the general approach, normally found in each type of disclosure.

It was evident that, both when regarding the preparation of a patent document, and that of a scientific article, it should address the naming of the object under study, by means of a title, the objectives of the research/ technology, besides contextualizing the problem to be investigated or treated.

On the other hand, it was clearly noted that the document of a patent application should cover the problem in a more objective manner, that is, in a more tactile and easily determined way, based on the fact that such a problem is related to equipment, processes, products, and the use of new technologies. Therefore, for the text of a patent, it is fundamental to relate the problem to its possible technical solutions.

As to the scientific publication, that is, from the academic viewpoint, the problem is often treated in a much broader fashion, and scientifically speaking, it may deal with something not necessarily considered a problem to some people. Academically approached problems may be, for example, enzymes not yet discovered or characterized, as well as metabolic processes not yet explained. Academic problems may refer to different interpretations of the same theme or to the same experiment carried out.

However, when the scientific publication is considered a result of the solution found for a problem not yet resolved, even so, it may distance itself from a patent application, since the word “problem” is interpreted, scientifically, as something not yet shown in practice. As a result, the solution of this type of problem may or may not pass through an inventive process. Sometimes, the solution to a problem occurs by the practical performance of experiments not yet carried out, but that, theoretically, are obvious consequences of lines of reasoning already previously outlined.

As to the structure of a patent document, it was also observed that this should be a descriptive report, at the time of application, composed of the items that define the field of invention, status of the technique, objectives, the invention in conceptual terms, as well as its advantages and benefits proposed in face of the technical solutions published in the status of the technique, besides the possible implementations of the invention, with the support of illustrations, when applicable.

Furthermore, besides the differences herein described, the careful preparation of a patent document should follow certain formal rules and principles, currently especially established by Normative Act 127 and LPI 9,279/96^(10)^, always observing not only the correct wording of the descriptive report, but also a careful evaluation and formulation of the scope of protection of the patent; many times, it is recommended that this step be considered and discussed in cooperation with a specialist in intellectual property.

Finally, it should be pointed out that a scientific article and a patent document offer, in their particularities, effective and comprehensive means for the best disclosure and protection, both of the national scientific production, and of the technological production of the country, in which the latter, many times, stems from scientific thinking applied in our laboratories and research centers. What should be carefully evaluated is that when a research project presents as a result a potentially inventive technical solution, due to an existing problem, the researchers, or participating inventors, should observe the crucial points of the technological development in order to better protect it, before the eventual publication, by means of a wellprepared descriptive report and claims chart, thus affording more robust and safe ways for future licensing and/or marketing of the new technology in partnership with the productive sector.
